# High antiretroviral therapy service delivery satisfaction and itsʼ associated factors at Midre-genet hospital; Northwest Tigray, Ethiopia

**DOI:** 10.1186/s12913-018-3055-4

**Published:** 2018-03-27

**Authors:** Kiflay Gebremariam Atsebeha, Daniel Haile Chercos

**Affiliations:** Department of Environmental and Occupational Health Safety, Institute of Public Health, University of Gondar, Gondar City, Ethiopia

**Keywords:** Patients satisfaction, ART, HIV/AIDS patients, Referral hospital

## Abstract

**Background:**

Patient’s satisfaction occurs when patients feel their needs and expectations are being meet by the service delivery they receive in health institutions. The Objective of this research was to assess HIV/AIDS patients’ satisfaction with antiretroviral therapy service delivery and its associated factors at Midre – Genet hospital, Northwest Tigray, Ethiopia**.**

**Methods:**

Institutional based cross-sectional study was conducted. Four hundred twenty study respondents were selected using systematic random sampling technique. Data was collected using pretested, structured interview questionnaire. Bivariate analysis was conducted to identify potential significantly associated variables and transferred to multivariate logistic regression analysis. *P* value < 0.05 is used to demarcate significantly associated variables.

**Result:**

The overall HIV/AIDS patients’satisfaction on antiretroviral therapy service delivery at Midre-Genet referral hospital was 75.2%. Multivariate analysis revealed age, marital status, occupation, income, information provision and guidance, privacy, access of toilets and interpersonal communication as significantly associated factors with patient’s satisfaction on ART service delivery.

**Conclusion:**

The overall HIV/AIDS patients’ satisfaction at Midre-Genet referral hospital was high. Age, marital status, occupation and, income was associated factors. Moreover, lack of functional CD4 machine, inaccessibility of toilets and water, waiting time, inadequate counseling services and lack of qualified health worker (Doctor) in the ART clinic were also challenges for ART patients in the hospital.

**Electronic supplementary material:**

The online version of this article (10.1186/s12913-018-3055-4) contains supplementary material, which is available to authorized users.

## Background

In 1981, the Centers for Disease Control and Prevention reported unusual clusters of *Pneumocystis carinii* pneumonia and Kaposi’s sarcoma in gay men in parts of the US. These were the first reported cases of Acquired Immune Deficiency Syndrome (AIDS) [1]. However, from all regions in the world, sub-Saharan Africa was the hardest hit by HIV pandemic, containing around 70% of people living with HIV/AIDS. More than 30 years later after the first cases were reported in 1981, there were 34.2 million people currently living with HIV and nearly 30 million people have died of AIDS related causes [2]. Moreover, the new modeling suggests that, compared with current treatment approaches, Treatment 2.0 (zero discrimination and zero AIDS related deaths) could avert an additional 10 million deaths by 2025 [3].

Ethiopia is one of the Sub-Saharan African countries with high level of patients with HIV/AIDS. [4, 5]. The government of Ethiopia responded to the HIV/AIDS epidemic as early as 1985. Consequently, Federal Ministry of Health (FMoH) of Ethiopia has been implementing a sector-wide reform to improve the quality and accessibility of ART care service in health institutions throughout the country [6]. Hence, Rapid expansion of the ART program provided an unprecedented opportunity to rapidly scale up HIV/AIDS prevention, care and treatment services [7]. As a result, free ART service was launched in public hospitals in 2005 and in primary health care centers in 2006 as part of the service scaling-up. Since then, the number of patients ever started ART is estimated to be 439,301, of which 317,443 were on the treatment since in 2013 [8]. This showed significant progress compared to the baseline of 13,000 ART patients in 2005/06 [9]. The number of ART providing facilities are also grown to 913 of which about 80% are in primary health care centers [8, 10]. In Tegray region specifically, there are about 56,900 people currently living with HIV/AIDS region, from which 43,600 are above the age of 15 years [11].

Patient’s satisfaction occurs when patients feel their needs and expectations are being meet by the service delivery they receive in health institutions. It is the patient’s perception of care received compared with the care expected [12]. Clients with different biopyschosocial background having different service demand and expectations need to be addressed adequately [13, 14]. Evaluating to what extent patients are satisfied with health services is clinically relevant, as satisfied patients are more likely to comply with treatment [15]. Otherwise unmet client needs can be problematic since it causes lack of confidence in the healthcare system and healthcare providers [16, 17]. To satisfy patients, organizations must focus on the delivery process itself and ensure the service transaction produces results to which the client is entitled for, in ways that meet the client’s expectations for service delivery [18, 19].

In Ethiopia, patient’s satisfaction studies suggest that patients complain about the quality of services given in health institutions. Patient satisfaction study done on Amhara region hospitals identified dissatisfaction on waiting hours during registration, visitation of doctors after registration, laboratory services, re-visiting of the doctor for evaluation with laboratory results and acquisition of drugs from the hospitals’ pharmacies [20]. This might be due to the fact that the health professional to population ratio of the country is very low compared to the WHO standards [21]. Similar studies done in Addis Ababa public and private hospitals also indicated the presence of general dissatisfaction with the availability and cleanness of latrine in the hospital [22] and government workers in particular were dissatisfied with given hospital services than non-government workers [23]. Moreover past studies suggest higher dissatisfaction of ART services in Tigray regional zone compared to similar regions in Ethiopia [24].

Even though ART service is being provided since 2005 in Ethiopia, ART delivery in Tigray Zonal hospitals has been low compared to other regional hospital in the country. Conducting this research provides a significant contribution to policy makers by indicating areas of weaknesses, enabling to take timely measures of adjustment, realigning to ensure a successful strategy to fight HIV/AIDS and encourage patient centered health service programs.

## Methods

### Objective of the study

To assess HIV/AIDS patients’satisfaction on antiretroviral therapy service delivery and identify its associated factors at Midre-genet hospital, Northwest Tigray, Ethiopia.

### Study design

An institutional based cross-sectional study was conducted from March to August 2013.

### Study area and period

The study was conducted in the Northwest of Tigray region, at Midre-Genet zonal referral Hospital, of Shire – Endaslassie, Ethiopia. According to the 2012 census, Shire – Endaslassie as a zone with a total population of 835,853. It is located 1037 KM to the Northwest from the capital city of Ethiopia, Addis Ababa [25]. This zone has 8 districts with a total of 217 health posts, 37 health centers, 3 private health centers and one referral hospital. Malaria, Tuberculosis, HIV/AIDS and Vein occlusion Liver Disease are major prevalent diseases in this zone.

### Source and study population

All HIV/AIDS patients visiting the hospital as an outpatient for ART service were the source population. All ART clients coming to the hospital as an outpatient for ART service during the study period were selected as study population**.**

### Inclusion and exclusion criteria

#### Inclusion criteria

Patients with HIV/AIDS who have been on ART and who were 18 years old and above are included in the study.

#### Exclusion criteria

HIV/AIDS patients under ART who were unable to speak, having difficulties of hearing and has a mental problems were excluded from the study.

### Sample size and sampling

For the quantitative section, the sample size was calculated using a single population proportion formula. It was calculated taking 95% confidence interval, marginal error of 5%, patient satisfaction as 50%, and addition of 10% of sample for contingency yielding the sample size of 422 HIV/AIDS patients. Systematic random sampling technique was used to select patients who visit the ART service delivery until the number of predetermined sample size was achieved.

For qualitative part of the study, focus group discussion was employed with 32 participants, patients and health care providers. An in-depth interview was conducted on 12 patients and key informants, continued until it reached saturation level. Purposive sampling technique was used to select study participants.

### Operational definitions

#### Patients/clients

People who are living with HIV/AIDS and visit to the hospital, for ART service.

#### Courtesy/respect

Treating patients with dignity or respect and greeting by health providers.

#### Interpersonal communication

Clarity, ease of understanding and acceptability of the information given by health service providers about the nature of the disease, required treatment and use of drugs need for regular follow-up to ART clients.

#### Patients’ satisfaction

Patients care received from ART service delivery points and staff; computed by adding the satisfaction parameters captured through the five point likert scale. Respondents who fall within the mean score and above are considered as satisfied and the rest as dissatisfied.

#### Service delivery points

The various stations found within the hospital where patients receive specific services (such as Rooms for registration, examination, laboratory, result and prescription and pharmacy).

#### Waiting time

The time between the clients’ arrival at the service delivery points and the time the client received the health service.

### Data collection tools and procedures

To assess patient satisfaction, a pre - tested structured standardized questionnaire were prepared from “Assessment Tool for Operational Standards of the Ethiopian Hospital Reform Implementation Guidelines (EHRIG)”. The data was collected using face to face interview (see Additional file 1). Data was collected by experienced four nurses and supervised by two senior health officers. Focus group discussion was mediated by the principal investigators using pre-prepared focus group discussion guide. Finally, the in-depth interview was also conducted by principal investigators.

### Data quality control

The questionnaire was developed first in English and translated to Amharic and back to English by language experts for validity. The data collectors were trained for 4 days about data collection tool, questioning technique and ethical issues. The questionnaire was pre - tested on 38 (10%) patients in another similar hospital setup. Problems encounter during data collection was dealt immediately. For the qualitative data collection, the focus group discussion continued until it reached saturation level with similar repeated answers. The completeness of the questionnaires was checked before data entry.

### Data processing and analysis

The collected data, was edited, cleaned and then entered in to EPI-INFO version 3.5.1 and exported to SPSS version 20 for analysis. Binary logistic regression and chi-square test was conducted to identify the association between the explanatory variables and outcome variables (patients’ satisfaction). From the bivariate analysis, variables with *p*-value < 0.2 was considered for multivariate logistic regressions. Variables that has *p* – value < 0.5 in multivariate analysis was considered as significantly associated variables.

Qualitative data was transcribed manually from the audiotape records during the sessions. The data was coded; categorized and finally analyzed using open code 3.6.2.0 for windows.

The respondents’ satisfaction score asked in Likert scale was averaged to create mean score of satisfaction. Then for analytical purposes, the mean score was considered as a cutoff point.

### Ethical consideration

Before data collection, ethical approval and ethical clearance letter obtained from Institutional Review Board (IRB) of University Of Gondar, College of Medicine and Health Sciences and submitted to regional health bureau and Midre- Genet referral hospital medical director office.

Written informed consent was also obtained from respondents during data collection after the purpose of the study was explained. Confidentiality of the data were maintained and assured by replacing the respondent name with identification number in data collection. Privacy during interview was also ensured.

## Results

### Socio demographic characteristics of the respondents

A total of 420 respondents were enrolled in this study with a response rate of 99.5%. Among the client’s 276(65.7%) were females and 299(71.2%) urban residents. The mean age of the clients was 36 ± 9.87 year (Table 1).

**Table 1:** Socio-demographic characterstics of respondents at Midre-Genet Hospital, Northwest of Tigray, 2013 (*N* = 420)

Characteristics	Satisfaction of clients on ART service delivery	
	Number (*n* = 420)	Percent (%)
Sex		
Male	144	34.3
Female	276	65.7
Age group		
18–25	63	15
26–35	158	37.6
36–45	101	24
46+	98	23.3
Marital status		
Single	155	36.9
Married	95	22.6
Divorced	64	15.2
Widowed	35	8.3
Separated	71	16.9
Educational status		
Unable to read and write	123	29.3
Primary	108	25.3
Secondary	98	23.3
Diploma and above	91	21.7
Occupation		
Government employee	49	11.7
Merchant	149	35.5
Farmer	77	18.3
Daily laborer	91	21.7
Without job	54	12.9
Address		
Urban	299	71.2
Rural	121	28.8
Monthly income		
No income	58	13.8
< 500	101	24
500–1000	169	40.2
1001^+^	92	21.9

### ART service delivery related characteristics of respondents

Waiting time is one of the variable used to measure patients’ satisfaction on ART service delivery. Majority of patients 393(93.6%) waited 30 min to 1 h before examination by health providers.

Highest dissatisfaction was also scored with respect to access of toilet 155(39.9%) and information provision 106(25.2%). Among the respondents who were requested to check their CD4 level, none of them were able to get CD4 machine in the hospital. However, 222 (52.6%) of patients were satisfied with the measures used to keep privacy during examination and with availability of drugs 223(53.1%) (Table 2).

**Table 2:** ART services delivery relatd characterstics of respondents at Midre-Genet Hospital, Northwest of Tigray, 2013 (*N* = 420)

Characteristics	Satisfaction of clients on ART service delivery	
	Number (*n* = 420)	Percent (%)
Information provision and guidance		
Strongly disagree	106	25.2
Disagree	114	27.2
Neutral	200	47.6
Courtesy/respect		
Disagree	19	4.5
Neutral	70	16.7
Disagree	203	48.3
Strongly disagree	128	30.5
Privacy		
Neutral	198	47.1
Disagree	222	52.9
Access of toilets		
Strongly disagree	155	36.9
Disagree	155	36.9
Neutral	110	26.2
Availability of drugs		
Neutral	49	11.7
Agree	224	53.3
Strongly agree	147	35
Interpersonal communication		
Neutral	33	7.9
Agree	164	39
Strongly agree	223	53.1
Willingness of professionals		
Strongly agree	14	3.3
Disagree	202	48.1
Neutral	204	48.6
Waiting time to get patient card		
< 30 min	96	22.9
30 min to 1 h	322	76.8
61 min to 2 h	2	0.5
Waiting time before examination		
< 30 min	23	5.4
30 min to 1 h	393	93.6
61 min to 2 h	4	1
Lab. Ordered to test CD4 level		
Yes	133	31.7
No	287	68.3
Getting CD4 machine to test CD4 level		
Not ordered	287	68.3
No	133	31.7

### Factors associated with satisfaction of clients on ART service delivery

Multivariate analysis revealed, age, marital status, occupation, income, information provision and guidance, privacy, access of toilets and interpersonal communication as significantly associated variables with patient’s satisfaction on ART service delivery (*p* < 0.05).

The results indicated that, study participant who belong to the age group of 36–45 years were found to be 91.9% times more likely to be satisfied than those who were in the age group of 18–26 years [AOR = 0.081, (95% CI: 0.013, 0.509)].

Respondents who were married found to be 5 times more likely to be satisfied with the service than those who were single [AOR = 5, (95% CI: 1.202, 20.832)] and respondent who were divorced were 87% more likely to be satisfied than those who were single [AOR = 0 .130, (95% CI: 0.020, 0.834)].

With regard to occupation, clients who were merchants, daily laborer and farmers were 94.7, 94.7 and 98.2% times more likely to be satisfied than government employee [AOR = 0.053, (95% CI: 0.009, 0.314)], [AOR = 0.053, (95% CI: 0.008, 0.338)], and [AOR = 0.018, (95% CI: 0.001, 0.223)], respectively.

Study participants who had monthly income 500–1000 ETB and more than 1000 ETB were found to be 99 and 96.9% more likely to be satisfied than those who had no monthly income [AOR = 0.010, (95% CI: 0.001, 0.107)], and [AOR = 0.031, (95% CI: 0.002, 0.407)], respectively.

Clients who fell neutral and disagreed to the information provision and guidance were 93 and 90.5% times more likely to be satisfied than those who were strongly disagree [AOR = 0.070, (95% CI: 0.015, 0.322)] and [AOR = 0.095, (95% CI: 0.027, 0.333)], respectively. Furthermore, patients who reported as neutral and disagreed with the access of toilets were 93.7 and 77.9% times more likely to be satisfied than those who reported as strongly disagree [AOR = 0.063, (95% CI: 0.011, 0.374)] and [AOR = 0.221, (95% CI: 0.065, 0.748)], respectively.

Study participants who respond as agreed to the measures taken to assure privacy were found 70% times more likely to be satisfied than those who reported as strongly disagree [AOR = 0.3, (95% CI: 0.094, 0.955)].

With respect to interpersonal communication, patients who strongly agree and agree were 94.3 and 98.9% times more likely to be satisfied than those neutral [AOR = 0.057, (95% CI: 0.010, 0.308)], and [AOR = 0.011, (95% CI: 0.002, 0.079)], respectively (Table 3).

**Table 3:** Multivariate analysis of factors associated with satisfacton of client on ART service delivery at Midre-Genet Hospital, Northwest of Tigray, 2013 (*N* = 420)

	ART service satisfaction		COR (95% CI)	AOR (95% CI
	Dissatisfied	Satisfied		
Age				
18–25	42(66.7%)	21(33.3%)		1
26–35	35(22.2%)	123(77.8%)	0.142(0.075, 0.271)	0.336(0.077, 1.463)
36–45	13(12.9%)	88(87.1%)	0.074(0.034, 0.162)	0.081(0.013, 0.509) ^*^
46^+^	14(14.3%)	84(87.7%)	0.083(0.039, 0.180)	0.223(0.038, 1.309)
Marital status				
Single	46(29.7%)	109(70.3%)		1
Married	37(38.9%)	58(61.1%)	1.512(0.883, 0.587)	5.004(1.202, 20.832) ^*^
Divorced	7(10.9%)	57(89.1%)	0.291(0.123, 0.686)	0.130(0.020, 0.834) ^*^
Widowed	7(20.0%)	28(80.0%)	0.592(0.242, 1.453)	7.937(0.653, 96.535)
Separated	7(9.9%)	64(90.1%)	0.259(0.110, 0.608)	2.507(0.393, 15.996)
Occupation				
Government	29(59.1%)	20(40.8%)		1
Merchant	6(4%)	143(96%)	0.029(0.011, 0.078)	0.053(0.009, 0.314) ^*^
Farmer	12(15.6%)	65(84.4%)	0.127(0.055, 0.295)	0.018(0.001, 0.223) ^*^
Daily laborer	19(20.9%)	72(79.1%)	0.182(0.085, 0.390)	0.053(0.008, 0.338) ^*^
Without job	38(70.4%)	16(29.6%)	1.638(0.724, 3.704)	0.165(0.017, 1.574)
Monthly income				
No income	38(65.5%)	20(34.5%)		1
< 500	39(38.6%)	62(61.4%)	0.331(0.169, 0.649)	0.204(0.029, 1.436)
500–1000	19(11.3%)	150(88.7%)	0.067(0.032, 0.137)	0.010(0.001, 0.107) ^*^
> 1001	8(8.7%)	84(91.3%)	0.050(0.020, 0.124)	0.031(0.002, 0.407) ^*^
Information Provision				
Strongly disagree	79(74.5%)	27(25.5%)		1
Disagree	19(16.7%)	95(83.3%)	0.068(0.035, 0.132)	0.095(0.027, 0.333) ^*^
Neutral	6(3%)	194(97%)	0.011(0.004, 0.027)	0.070(0.015, 0.322) ^*^
Privacy				
Neutral	73(36.9%)	125(63.1%)		1
Agreed	31(14%)	191(86%)	0.278(0.173, 0.448)	0.300(0.094, 0.955) ^*^
Access of toilets				
Strongly disagree	84(54.2%)	71(45.8%)		1
Disagree	17(11.0%)	138(89.0%)	0.104(0.057, 0.189)	0.221(0.065, 0.748) ^*^
Neutral	3(2.7%)	107(97.3%)	0.024(0.007, 0.078)	0.063(0.011, 0.374) ^*^
Interpersonal communication				
Neutral	21(63.6%)	12(36.4%)		1
Agree	57(34.8%)	107(65.2%)	0.304(0.140, 0.663)	0.057(0.010, 0.308) ^*^
Strongly agree	26(11.7%)	197(88.3%)	0.075(0.033, 0.171)	0.011(0.002, 0.079) ^*^
Willingness of professionals				
Strongly disagree	7(50%)	7(50%)		1
Disagree	82(40.6%)	120(59.4%)	0.683(0.231, 2.022)	2.255(0.178, 28.610)
Neutral	15(7.3%)	189(92.7%)	0.079(0.025, 0.256)	0.476(0.033, 6.913)

Note: 1:00 = Reference

*Significant at *P* value < 0.05

## Discussion

The findings of this study revealed that the overall HIV/AIDS patients’satisfaction on antiretroviral therapy service delivery at Midre-Genet referral hospital was 75.2%. This finding is similar with the study done in Trinidad and Tobago which showed 75% level of satisfaction [26], but lower than findings, in Kuwait (99.6%) [27] and Addis Ababa (85%) [22]. Possible reasons might be better diagnostic facilities, good infrastructure, and qualified and adequate number of health professionals. However, this finding is higher than studies conducted in, Bangladesh (68%),Vietnam (42.4%), Jima university hospital, Ethiopia (57.1%), [28], Central Ethiopia(54.1%) [29], and Amhara region hospitals (22 to 50%) [20, 30–32]. Reasons might be due to efforts made to improve the service delivery process, availability and access of free drugs. The finding is also supported from the qualitative study where most of study participants in their focus group discussion have mention better and improved service delivery process than previous years, especially on the availability of drugs and supplies, welcoming, respect, close relationship given to ART clients, and the separation of ART clinic from the hospital which has increased their privacy. An in-depth interview with health providers also indicated that majority of clients were satisfied with the services provided at the ART clinic.

From this study, age groups of 36–45 years were found to be 91.9% times more likely to be satisfied than those who were in the age group of 18–25 years. This is similar with other studies conducted in Vietnam where older people are 99% times likely to be satisfied with the different services than the young ages [30]. Study done at Jimma specialized and teaching hospital, Ethiopia, also showed level of satisfaction score was directly related to age [32]. This could be due to the fact that as age increases, interpersonal relationship among clients and health providers improved.

The present study result revealed married and divorced respondents were found to be 5 and 87% times more likely to be satisfied than their single counterparts, respectively. A study from Vietnam also indicated that the satisfaction levels of single participants were decreased by an average of 0.0314 times towards the service delivery [30]. In Ethiopia, ART service delivery study from six hospitals reported that married study participants were 59% times more satisfied than the single ones. This might be due to the fact that married clients would have a support and care from their partners [33].

With respect to occupation, respondents of this study who were merchants, daily laborer and farmers were 94.7, 94.7 and 98.2% times more likely to be satisfied than government employee, respectively. This findings are higher than the study from public and private hospitals in Addis Ababa which reported that merchants, daily laborers and farmers were, 69.5, 86.5 and 84.6% more likely to be satisfied than government employee, respectively [23]. Additionally study conducted in Vietnam reported that unemployed respondents were 82% more likely to be satisfied than government employee [30]. This could be due to in partial that, ART services are provided in working hours and government employees might have shortage of time and have the tendency to have the service in short period of time. In addition, government employees most likely have high level of education in which a study done in Trinidad and Tobago shown that as level of education increase satisfaction of clients with the services delivered is decreased [26].

From this study, participants who had monthly income 500–1000 ETB and more than 1000 ETB were found to be 99 and 96.9% more likely to be satisfied than those who did not have monthly income. These findings are in line with the study done on governmental hospitals of Addis Ababa, Ethiopia indicating that clients who had monthly income above 491 ETB were about 2.25 times more likely to have improved quality of life and satisfied with the services than those who had income below 150 ETB [34]. The finding is also similar with the study done in Vietnam where, the richest clients were 81% more likely to be satisfied than the poorest one [30].

Clients who fell neutral and disagreed to the information provision and guidance towards the service delivery were 93 and 90.5% times more likely to satisfy than those who were strongly disagree. Study from Jimma hospital, Ethiopia, shown that information provision and guidance towards the service delivery was one of the factors for dissatisfaction [32]. Most of the clients in the focus group discussion also raised the issue of information provision and guidance by pointing out the absence of written information indicators towards the different service delivery points, even for those who can read. Key informants in the in-depth interview also admitted the absence of any written information available to ART clinic patients.

Study participants who reported as neutral and disagreed with the access of toilets were 93.7 and 77.9% times more likely to be satisfied than those who reported as strongly disagree, respectively. Lower satisfaction toward provision of safe and clean toilet to patients was observed from a study done in Bangladesh which showed that satisfaction with cleanness of toilet was only 54%. In addition, satisfaction with accessibility and availability of latrines in Addis Ababa, Ethiopia, was only 64.5%; which affect satisfaction of clients [22, 31]. Most of respondents from their FGD of this study stated the absence of toilet around the ART clinic, the already existed toilet of the hospital is too dirty, and as some result clients were forced to use the open defecation in the hospital compound.

Study participants who respond as agreed to the measures taken to assure privacy were found 70% times more likely to be satisfied than those who reported as strongly disagree. Similar findings were observed from Muhimbili hospitals in Dare Salaam where, majority of patients (81.6%) who did not have privacy during consultation with their doctor had lower satisfaction [35].

Regarding to interpersonal communication, patients who were strongly agree and agree were 94.3 and 98.9% times more likely to be satisfied than the neutral. This is in agreement with the study conducted at health centers of central Ethiopia where health service providers who had excellent communication had an average increase of 44% satisfaction score compared to those who had good communication [29].

With respect to waiting time, this study showed that 93.6 and 76.7% respondents had to wait 30 min to 1 h before examination by health providers and to get their patient card, respectively. This was higher than the study done at Jimma university hospital, Ethiopia where, 80% and more than 50% clients were to be served within 15 min, at the card room and at the ART clinic respectively [32]. This is also higher than with the study report from London which indicates 47% of the respondents were served by their doctors within 15 min [36]. Study conducted on laboratory services in Addis Ababa Ethiopia, shown clients who waited less than 30 min were 7.5 times more likely to be satisfied with the service delivery than those waited more than 30 min [22]. Most respondents in FGD had also explained they killed their time waiting for about 1 h to be seen by the doctor at the hospital together with the other patients and mentioned problems associated with card systems.

With respect to the CD4 machine, among the clients who were requested to check their CD4 level (133 respondents) none of them were able to get the CD4 machine to test their CD4 cell count. Study report on satisfaction with laboratory services from Addis Ababa, Ethiopia shown that, respondents who had all ordered laboratory tests were 2.36 times likely to be satisfied than those who did not had the laboratory service. Most of the respondents participated in the FGD had also explained the problems related with nonfunctional CD4 machine at the hospital for more than 2 years and patients were referred to Axum hospital to check their CD4 cell count. An in-depth interview with key personnel supported the problem of CD4 machine and pointed out the big impact on the satisfaction of patients with respect to the service.

## Conclusion

The overall HIV/AIDS patients’satisfaction on antiretroviral therapy service delivery at Midre-Genet hospital, Northwest of Tigray, was high as compared to similar studies conducted previously in Ethiopia. Age, marital status, occupation, income, information provision and guidance, privacy, access of toilets and interpersonal communication were found to determine HIV/AIDS patients’ satisfaction. Based on the finding of this study, authors recommend to Midre-Genet Hospital to increase professional to reduce the waiting time, provide long and short-term training for professional working at ART clinic on effective interpersonal communication, work on provision of private examination rooms for ART patients, and provide clean and adequate toilets and water to ART patients.

## Additional file

Additional file 1: English version of data collection tools. All the data collection tools used in this research which includes structured questionnaires, focus group discussion outlines and outline for in-depth interview. (DOCX 22 kb)

## Abbreviations

AIDS: Acquired Immuno-Deficiency Syndrome
AOR: Adjusted odds ratio
ART: Antiretroviral therapy
ARV: Antiretroviral
FMOH: Federal Ministry of Health
HAPCO: HIV/AIDS prevention and control office
HIV: Human Immuno-Deficiency virus
HSDP: Health Sector development Plan
PLHA: People Living With HIV/AIDS
UNAIDS: Joint United Nations Programme on HIV/AIDS
WHO: World Health Organization
