# Supplementary material for: High antiretroviral therapy service delivery satisfaction and itsʼ associated factors at Midre-genet hospital; Northwest Tigray, Ethiopia
Source: BMC Health Serv Res. 2018 Mar 27;18:223. doi: 10.1186/s12913-018-3055-4 (PMC5872538; doi:10.1186/s12913-018-3055-4)
Supplement: Supplementary file 1 — English version of data collection tools. All the data collection tools used in this research which includes structured questionnaires, focus group discussion outlines and outline for in-depth interview. (DOCX 22 kb) [file 12913_2018_3055_MOESM1_ESM.docx]

**English version of data collection tools**

## English version information sheet and consent form

## Information sheet

Name of Investigators: Kiflay G/mariam and Daniel Haile Chercos

Name of organization: University of Gondar College of medicine and Health science and institute of public health.

Introduction: This information sheet & consent form is prepared by the principal investigators whose aim is to study assessment of patients’ satisfaction for HIV/AIDS on ART treatment and associated factors. Others that will be participated in this research include two trained senior health officer supervisors, and four Nurses for data collection.

**Purpose of the research project**: Assessment of patient’s satisfaction for HIV/AID on ART service delivery and associated factors. Different studies conducted in the country indicated that patient satisfaction for HIV/ADS on ART service delivery in Tigray is low. Therefore this study will help to identify the problems encountered during service delivery for HIV/AIDS treatment and related factors which is necessary for the improvement of level of satisfaction at the study hospital, regionally as well as nationally.

Procedure: This study will be conducted mainly on HIV/AIDS patients who are on ART. Before starting the research, Ethical clearance will be obtained from university of Gondar ethical review board and a permission letter from Tigray Regional Health Bureau, Northwest Tigray Health office, Medical director of Midre-Genet hospital, and written consent from the study participants will also be obtained. At last, ART patient will be interviewed with structured questioner and relevant bodies will be participated in guided focus group discussion.

Risk and or discomfort: by participating in this research project you will not feel any discomfort and the interview will last only 20 to 30 minutes. Information gathered will be kept confidently. There is no risk at all by participating in this research.

Benefits: your participation in this research project, may not give you a direct benefit. But it has great role to achieve improvements in the ART service delivery.

Incentives: you will not get paid or have any other incentives to take part in this project.

**Confidentiality and anonymity:** The information we collate form this research will be kept confidential. Information about you that will be collected from this study will be stored in a file, which will not have your name on it. The investigator will store by locking so that unintended body will not have accesses to it. Your name will not be written on this form & used in connection with any of the information you tell me.

Right to refuse and/ withdraw: you have full right to refuse form participating in this research you can choose not to respond some or all of the questions. This will not affect your health service delivery that you got form the hospital.

## Consent form:

Hello! My name is ______________________I am working in the research team as a postgraduate student in the University of Gondar, College of Medicine and Health Science, and Institute of Public Health. I would like to gather information regarding the level of patients’ health care satisfaction with antiretroviral treatment service delivery system while you are in this health institution which is very important to improve the service delivery. Your cooperation and willingness to give the information is helpful in identifying problems related to the concern. We have identified you as a study participant believing that you would be willing to respond to questions to give me your sincere and truthful answers. By participating in this research project, your name or address will never be written in this form. All information that you give will be kept strictly confidential. Your participation is voluntary and you are not obliged to answer any question you do not wish to answer. If you have any question or anything unclear, please feel free to ask. If you are not still comfortable along the discussion process you have full right to drop it any time you want. You will not be asked to give reasons and you will not miss any chance of service delivery from the hospital for doing so.

If you are clear with the information provided, do I have your permission to continue?

1. If yes_______________ thank you, continue to the next page.
2. If no________________ thank you, stop.

# Structured English Version Questionnaire

## University of Gondar

## College of Medicine and Health Sciences

## Institute of Public Health

Questionnaire developed for data collection of patients’ satisfaction on antiretroviral therapy service delivery at Midre-genet hospital, Shire – Endaselassie, 2013

### Identification:

Name of the health institution_______________________ Code No.___________

Date of interview________________ time started ________time finished_______

Interviewer or supervisor who certified that, informed consent has been given from the respondents.

Name of interviewer _________________________ Signature ____________

Name of supervisor __________________________ signature _____________

### Result code

1. Completed

2. Refused

3. Partially completed

# Part 1: Background information on Socio-Demographic Factors

Instruction: You can circle the appropriate one

| Serial number. |  |
| --- | --- |
| 101. Sex | 1. Male 2. Female |
| 102. How old are you? | _______ years old |
| 103. What is your Marital Status | 1. Single 2. Married 3. Divorced 4. Widowed 5. Separated |
| 104. Educational Status | 1. Unable to read and write  2. Elementary (1-8)  3. High school to preparatory(9-12)  4. Diploma  5. Degree and above |
| 105. Occupation | 1. Governmental employee  2. Merchant  3. Farmer  4. Daily laborer  5. without job  6. Others |
| 106. Address | 1. Urban 2. Rural |
| 107. Monthly income (per individual) | __________ETH Dollars |

**Part 2**. Information on Health service delivery related factors:

| 108. Did you feel that the schedule of service  Hours at the hospital was convenient for you? | | 1. Yes  2. No | |
| --- | --- | --- | --- |
|  | | | |
| 109. You get adequate provision of information and guidance to the different services of the hospital? (In locating for registration rooms, examination rooms, laboratory, pharmacy, toilet etc.). | | | 1. Strongly disagree  2. Disagree  3. Neutral  4. Agree  5. Strongly agree |
| 110. How long did you wait to get your card in the health institution’s card room? | | | _______minutes/hour |
| 111. Most of the time how long did you wait before seeing a Doctor/health worker? | | | _______minutes/hour |
| 112. You feel satisfied with the time spent waiting to be seen by a health worker? | | | 1. Strongly disagree  2. Disagree  3. Neutral  4. Agree  5. Strongly agree |
| 113. You are served with courtesy and respect of the Doctor/Nurse during your visit? | | | 1. Strongly disagree  2. Disagree  3. Neutral  4. Agree  5. Strongly agree |
| 114. Doctor/nurses are willing to answer your questions about your health problem? | | | 1. Strongly disagree  2. Disagree  3. Neutral  4 .Agree  5. Strongly agree |
| 115. The necessary measures were taken to  Assure your privacy during examinations? For  example, a private room, screened area, etc | | | 1. Strongly disagree  2. Disagree  3. Neutral  4. Agree  5. Strongly agree |
| 116. Were any Laboratory procedures ordered to check your CD4 level?  → If no go to question number 123. | | | 1. Yes  2. No |
| 117. If yes, did you get the ordered CD4 machine for  Laboratory in the hospital? | | | 1. Yes  2. No |
| 118. If yes, how long did you wait to give specimen for the  Lab? | | | _______minutes/hour |
| 120. How long did you wait to see the Doctor after receiving your results? | | | _______minutes/hour |
| 121. You have an access and availability of toilets around the ART clinic? | | | 1. Strongly disagree  2. Disagree  3. Neutral  4. Agree  5. Strongly agree |
| 122. Were the toilets regularly cleaned? | | | 1. yes  2. no |
| 124. Were drugs and supplies ordered to you?  → If no go to question number 129. | | | 1. Yes  2. No |
| 125. Do you agree that you get all the prescribed drugs  and supplies ordered to you without any fee in the  drug dispensary room/ pharmacy of the ART clinic or hospital? | | | 1. Strongly disagree  2. Disagree  3. Neutral  4. Agree  5. Strongly agree |
| 126. You believe that the information given to you about  your health problem by the health providers is  complete? | | | 1. Strongly disagree  2. Disagree  3. Neutral  4. Agree  5. Strongly agre |
| 127. During your communication with the doctor/nurse, the information given to you is clear and easily understood. (About the nature of the disease, treatment requirement, advices e.t.c.). | | | 1. Strongly disagree  2. Disagree  3. Neutral  4. Agree  5. Strongly agree |
| 128. you are satisfied with the professional’s  explanation of medical terms to you? | | | 1. Strongly disagree  2. Disagree  3. Neutral  4. Agree  5. Strongly agree |
| 129. Could you recommend the services of this hospital  to Someone else? | | | 1. Yes  2. No |
| 130. On a scale of 0-10 (0 being the  worst facility, 10 being the best  facility), how would you rate this  health facility?: | 􀂉 􀂉 􀂉 􀂉 􀂉 􀂉 􀂉 􀂉 􀂉 􀂉 􀂉 0 1 2 3 4 5 6 7 8 9 10  Worst………..…………………….………….Best | | |

# Guiding questions for Focus Group Discussion

Focus group discussion for patients’ satisfaction with ART service delivery at Madre-Genet hospital.

1. How do you explain the “ART service delivery”?
2. How do you explain your satisfaction on ART service?
3. What are the factors that affect your satisfaction?
4. How common/rare is missing ART drugs among PLHA on ART?
5. What are the reasons for missing ART drugs?
6. How do you express the courtesy and respect given to you by the Dr/health workers? at this hospital?
7. Do you get full laboratory and x ray services in the hospital?
8. Do you get the prescribed drugs and supplies in the hospital?
9. Can you please tell me about the waiting time to get the services of ART in the hospital?
10. What should be done to improve the satisfaction of clients on ART Service delivery at this hospital?

**Guideline for in-depth interview**

1. How do you explain the ART Service delivery at this hospital?

2. How do you explain client's satisfaction receiving ART services at this hospital?

3. How common/rare is missing ART drugs in this hospital?

4. What could be the possible reasons for clients missing ART drugs in this hospital?

5. What do you suggest to improve client's satisfaction at this hospital?

6. Do you have any additional comment on satisfaction of ART service delivery?
